# Network analysis of work-family support and career identity and their associations with job burnout among primary healthcare workers: a cross-sectional study

**DOI:** 10.3389/fpubh.2025.1581624

**Published:** 2025-06-26

**Authors:** Si-cheng Liu, Yuan Xu, Ming Yang, Jia-yi Sun, Qi-rong Qin, Gui-xia Fang

**Affiliations:** ^1^School of Health Management, Anhui Medical University, Hefei, China; ^2^Department of Epidemiology and Biostatistics, School of Public Health, Anhui Medical University, Hefei, Anhui, China; ^3^Ma’anshan Center for Disease Control and Prevention (Ma’anshan Health Supervision Institute), Ma’anshan, China; ^4^The Affiliated Ma’anshan Center for Disease Control and Prevention of Anhui Medical University, Ma’anshan, China; ^5^Institute of Hospital Management, Anhui Medical University, Hefei, Anhui, China

**Keywords:** job burnout, career identity, work-family support, scientific perspective, propensity score matching, primary healthcare, network structure

## Abstract

**Objective:**

To explore the complex associations between job burnout, career identity, and work-family support among primary healthcare workers from a network perspective.

**Methods:**

Data were sourced from primary healthcare institutions in China’s central provinces. We used the Maslach Burnout Inventory Comprehensive Survey, a career identity scale tailored for primary healthcare workers, and the Chinese version of the Work-Family Support Questionnaire. A Gaussian network model was used to identify key factors, with “central nodes” being those that strongly influence others and “bridge nodes” connecting different parts of the network.

**Results:**

Of the 8,135 participants surveyed, 5,120 (62.9%) reported job burnout. Compared to those with burnout, the non-burnout group scored higher in career identity, family support, and work support (54.29 vs. 49.42; 71.58 vs. 61.26; 35.03 vs. 31.20; *p* < 0.001). Network analysis revealed structural differences in the burnout-support-identity networks between groups after propensity score matching (M = 0.261, *p* < 0.001). In both groups, “understanding of role, content and requirements” were central nodes, while “work and family support” served as bridge nodes. Burnout was closely related to work support, family support, job suitability, and media criticism.

**Conclusion:**

Targeting central and bridge nodes can help reduce job burnout among primary healthcare workers.

## 1 Introduction

In recent years, the growing number of older individuals and increasing rates of chronic illnesses have intensified the necessity for novel primary healthcare frameworks (1, 2). The comprehensive reform of primary healthcare continues to advance worldwide. However, this progress has amplified the burden of responsibilities and tasks on practitioners (3, 4). As the demand for high-quality primary healthcare services increases, the expectations of practitioners have escalated, potentially exacerbating instances of burnout, a critical challenge that threatens workforce sustainability and service quality.

Burnout, a psychological and physical response to prolonged, unmanaged, work-related stress, is characterized by emotional exhaustion, depersonalization, and reduced professional efficacy (5). This phenomenon can be attributed to various factors, particularly in high-stress work environments (6). Globally, burnout prevalence among healthcare workers is alarming; 40% of US physicians report at least one symptom (7), while one-third of UK medical trainees experience severe burnout (8). This phenomenon is particularly detrimental to career identity, a construct reflecting an individual’s positive evaluation of their profession, emotional attachment and alignment of self-concept with work (9, 10).

While burnout is widely linked to an eroded career identity, emerging evidence suggests resilience pathways. For instance, dual support systems (organizational and familial) mitigate burnout and bolster career identity among medical staff (11), highlighting the interplay between external resources and internal psychological states. Such findings align with the Conservation of Resources (COR) theory (12), which posits that individuals strive to retain and protect resources (e.g., social support) and that resource loss exacerbates stress, whereas resource gain fosters resilience. Critically, differences in individuals’ abilities to successfully adapt to adversity through the dynamic interaction between personal traits and environmental resources modulate this resource mobilization process (13). Individuals with high levels of resilience exhibit an enhanced ability to transform external support into psychological capital (14). Similarly, the Job Demands-Resources (JD-R) model frames burnout as a consequence of imbalanced demands and resources, with career identity acting as a motivational outcome shaped by resource availability (15). These theoretical lenses underscore the need to examine how situational variables (e.g., work-family support) and dispositional factors (e.g., career identity) interact dynamically in burnout contexts.

Despite this progress, critical gaps remain. First, previous studies have often isolated burnout, support systems, or career identity, neglecting their interconnectedness. For example, although social support buffers burnout (16, 17), its role in sustaining career identity through resource replenishment remains underexplored. Second, methodological limitations such as reliance on linear models fail to capture the complex nonlinear relationships among these constructs. Network Analysis has emerged as a transformative tool. Rooted in systems theory (18), network analysis conceptualizes psychological constructs as dynamic systems of interacting elements where nodes influence one another directly and indirectly (19, 20). Unlike traditional methods, this approach identifies central nodes (highly influential variables) and bridge nodes (key nodes connecting different parts of a network), offering actionable insights for targeted interventions (21, 22). For instance, network studies revealed “professional self-concept” as a bridge between coping strategies and career identity in nursing students (23), and “guilt” as a critical link between depression and suicidality in healthcare workers post-pandemic (24). However, no research has applied this framework to unravel the burnout-support-identity network in primary healthcare workers—a gap this study addresses. Notably, network approaches align with contemporary resilience frameworks that conceptualize adaptation as an emergent property of interconnected bio-psycho-social systems (25). By mapping how specific resilience components (e.g., emotional regulation and purposefulness) occupy central positions in the burnout-support-identity network, we can identify potent intervention targets that maximize individual differences in stress adaptation.

This study explored the complex relationships between job burnout, career identity, and work-family support among primary healthcare workers from a network perspective. By integrating COR and JD-R theories with network science, this research advances a novel framework for understanding how support systems shape self-perception under chronic stress. Practically, it provides tailored strategies to enhance support structures, reinforce career identity, and improve workforce resilience and healthcare quality in primary care settings.

## 2 Methods

### 2.1 Research questions and hypotheses

Based on the COR theory and network resilience perspectives, we formulated three research questions (RQs) with the following hypotheses:

RQ1: How do burnout-support-identity network structures differ between primary healthcare workers with and without burnout?

*H*1: Burnout networks exhibit stronger negative connections between support systems and career identity nodes than non-burnout networks. According to the COR theory, resource loss spirals exacerbate stress pathways, leading to intensified negative interactions between support systems and career identity. As individuals experience burnout, the depletion of resources such as emotional and social support contributes to a weakening of their professional identity, creating a vicious cycle that further deepens stress and burnout symptoms.

RQ2: Which nodes act as critical bridges linking burnout symptoms to support systems and career identities?

*H*2: Work support is the most influential bridge node in both groups; however, depersonalization gains bridge centrality in burnout networks. Tawfik et al. (5) reported that depersonalization mediates 68% of the effects between support systems and burnout, highlighting its critical role as a bridge node. This finding demonstrates that depersonalization not only reflects but actively contributes to the breakdown of supportive relationships and career identity in burnout scenarios.

RQ3: What are the dominant pathways through which burnout propagates in the integrated system?

*H*3: Occupational burnout primarily spreads through emotional exhaustion, weakening of work support, and a decline in professional identity. Longitudinal studies have shown that emotional exhaustion precedes the deterioration of support systems by 3–6 months, as evidenced by research conducted by various scholars (26). These findings highlight the temporal sequence in which burnout symptoms unfold and provide a clear roadmap of how emotional exhaustion leads to the erosion of work support and career identity over time.

### 2.2 Participants and procedures

From March to May 2022, a multistage sampling method was employed to select participants from a province in Central China, and 320 primary healthcare facilities were identified. Following communication with the survey sites, electronic questionnaires were distributed by the head of the municipal health department in collaboration with the “WeChat Questionnaire Star.” Cluster sampling was used to assess primary healthcare workers, including general practitioners, nurses, public health physicians, pharmacists, and others who met the inclusion criteria. The inclusion criteria were as follows: (1) a minimum of 1 year of experience in primary healthcare and (2) provision of informed consent and agreement to voluntarily participate in this study (27). 8,339 primary healthcare workers were invited to participate in the survey, of whom 8,135 met the inclusion criteria and were included in the analyses. The surveys were conducted anonymously. All procedures adhered to the ethical standards established by the Committee of the Anhui Medical University (No. 83220442).

### 2.3 Measurements

#### 2.3.1 Demographic characteristics

Data gathered on the sociodemographic factors and job characteristics of primary healthcare workers included information on sex, age, educational background, job title, years of experience, typical annual salary, and average daily working hours.

#### 2.3.2 Scales and questionnaires

The Maslach Burnout Inventory Comprehensive Survey (MBI-GS) was used in this study to measure the job burnout levels of primary healthcare workers. This inventory comprises of 15 items categorized into three dimensions: emotional exhaustion (EE, five items), depersonalization (DP, four items), and low personal achievement (LPA, six items). Responses were assessed using a seven-point Likert scale ranging from zero (never) to six (every day). Notably, LPA scored in the opposite direction, whereas the other dimensions scored positively. The score for each dimension of the MBI-GS was calculated as the mean of the respective item scores, and overall burnout composite score was determined using the formula 0.4*EE + 0.3*DP + 0.3*LPA. The total score ranged from zero to six. A composite score of less than 1.5 indicated the absence of burnout, whereas a score of 1.5 or higher signified the presence of burnout (28).

The Work-Family Support Questionnaire developed by Li et al. (29) is suitable for Chinese employees. After exploratory and confirmatory factor analyses, 26 items were determined in two dimensions: work-domain support (18 items) and family domain support (eight items). Each item was scored on a five-point Likert scale ranging from one (strongly disagree) to five (strongly agree). The total score on the Work-family Support scale ranged from 26 to 130, with higher scores indicating higher levels of work-family support among primary healthcare workers.

With reference to the Nurse Professional Identity Scale, translated and validated by Liu et al. (30), a career identity scale suitable for primary healthcare workers was developed after discussions between the subject group and experts. The scale consists of 12 items and includes two dimensions: a sense of coherence and a sense of meaningfulness. Each item is rated on a five-point Likert scale ranging from one (strongly disagree) to five (strongly agree). The total score of the scale ranged from 12 to 60, and the higher the score, the stronger the career identity of the primary healthcare workers. Regarding the potential conceptual overlap between the MBI-GS and the professional identity scale, particularly in the area of personal accomplishment, personal accomplishment in the MBI-GS is typically related to job performance and self-efficacy (31, 32). In contrast, the professional identity developed in this study focuses more on an individual’s sense of identification with and belonging to their professional role. Previous studies using the MBI have found that burnout acts as a full mediator between professional identity and turnover intention (33), ensuring their effectiveness as independent nodes in the research. In this study, Cronbach’s alpha coefficients of the MBI-GS, Work-Family Support Questionnaire, and Nurse Professional Identity Scale were 0.88, 0.97, and 0.90, respectively.

### 2.4 Data analysis

Descriptive analysis was performed using SPSS version 23.0, and network analysis was performed using RStudio with R version 4.2.0. To address and minimize potential confounding biases, individuals diagnosed with burnout were systematically matched with individuals without burnout using a 1:1 Propensity Score Matching (PSM) technique. A caliper value of 0.2, which was obtained using the R Matching package. The R-qGraph package was used to determine the network structure. Our approach combined a Gaussian graphical model that effectively integrates graphical lasso techniques with an extended Bayesian information criterion (34). Gaussian network models are commonly used to describe the relationship between continuous variables, where the relationship between variables is represented by a covariance matrix.

The Network Comparison Test (NCT) function was used to analyze the differences between networks characterized by burnout and non-burnout traits. In the Gaussian network model, each node represents a random variable and the edges between nodes represent the correlation between them (35). The expected impact of each node within the network was assessed using the qGraph software. An increase in the expected impact indicated that these nodes played a critical role in the network. In addition, we used the R package mgm to assess the predictability of each node, which refers to the extent to which a node can be predicted by the state or behavior of its neighboring nodes. This predictability helps us understand the mutual influence and dependency between nodes in the network.

The BootNet package was used to evaluate the accuracy and dependability of the networks. To evaluate the accuracy of the edge weights, we used the edge-weight bootstrap technique to calculate 95% confidence intervals (CIs). A larger overlap in CIs indicates a higher level of accuracy. Using the case-dropping bootstrap method, we evaluated the stability of node importance or centrality within the network. The centrality stability coefficient was used as an indicator of stability. To measure the stability of the centrality indices, we calculated the correlation stability coefficient for correlation values that were equal to or greater than *r* = 0.7. A coefficient value of 0.25 or more is deemed acceptable, whereas a value of 0.5 or above is viewed as excellent. The flow network describes the critical issues that determine network functioning. By analyzing the maximum flow capacity of each edge, which represents the maximum capacity limit of the edge, we can calculate the importance of each node within the network model. In this study, we investigated the process network of samples that have a comprehensive burnout score of 1.5 or higher, namely the group experiencing job burnout.

## 3 Results

### 3.1 Participants information

The positive rate of job burnout was 62.9%, 5,120 respondents had burnout, and 3,015 respondents did not. The demographic characteristics are shown in Table 1. After PSM, 2,986 primary healthcare workers with burnout and an equal number of non-burnout counterparts were included in the analysis. No significant differences were detected between the two groups in terms of age, sex, workplace location, educational background, professional qualifications, work tenure, daily working hours, and overtime frequency. However, the total scores of career identity, work support, and family support for the burnout group were inferior to those of the non-burnout group (49.49 ± 6.0 vs. 54.31 ± 5.2; 62.02 ± 14.5 vs. 71.58 ± 13.8; 31.34 ± 5.5 vs. 35.04 ± 4.9) and all the *p*-values were less than 0.001. The detailed population characteristics after pairing are presented in Table 2.

**Table 1:** Descriptive characteristics of 8,135 participants.

Characteristic	Total	Burnout status		*p*
		Non-burnout	Burnout	
Age (N, %)				<0.001
≤25	509(6.3)	136(4.5)	373(7.3)	
26–35	2,319(28.5)	668(22.2)	1,651(32.2)	
36–45	2,608(32.1)	1,049(34.8)	1,559(30.4)	
46–55	2,386(29.3)	1,034(34.3)	1,352(26.4)	
≥56	313(3.8)	128(4.2)	185(3.6)	
Gender (N, %)				0.686
Male	2,618(32.2)	979(32.5)	1,639(32.0)	
Female	5,517(67.8)	2036(67.5)	3,481(68.0)	
Office address (N, %)				0.368
Community	2,454(30.2)	928(30.8)	1,526(29.8)	
Township	5,681(69.8)	2087(62.9)	3,594(70.2)	
Education (N, %)				<0.001
High school and below	1,636(20.1)	710(23.5)	926(18.1)	
Vocational school	3,504(43.1)	1,313(43.5)	2,191(42.8)	
Associate degree and above	2,995(36.8)	992(32.9)	2003(39.1)	
Professional title (N, %)				0.145
Intermediate and above	2,815(34.6)	1,076(35.7)	1739(34.0)	
Entry	4,467(54.9)	1,613(53.5)	2,854(55.7)	
Senior	853(10.5)	326(10.8)	527(10.3)	
Working experience (N, %)				<0.001
≤10 years	2,675(32.9)	811(26.9)	1864(36.4)	
11 to 20 years	2029(24.9)	743(24.6)	1,286(25.1)	
≥21 years	3,431(42.2)	1,461(48.5)	1970(38.5)	
Annual income (N, %)				0.776
<30,000	1,257(15.5)	459(15.2)	798(15.6)	
30,000–50,000	3,059(37.6)	1,148(38.1)	1911(37.3)	
>50,000	3,819(46.9)	1,408(46.7)	2,411(47.1)	
Daily working hours (N, %)				<0.001
<8	3,882(47.7)	1,524(50.5)	2,358(46.1)	
8–10	3,133(38.5)	1,106(36.7)	2027(39.6)	
>10	1,120(13.8)	385(12.8)	735(14.4)	
Overtime frequency (N, %)				<0.001
No overtime required	1,419(17.4)	645(21.4)	774(15.1)	
Occasional overtime	4,598(56.6)	1724(57.2)	2,874(56.1)	
Frequent overtime	2,118(26.0)	646(21.4)	1,472(28.7)	
Career Identity score (mean±SD)	51.23 ± 6.2	54.29 ± 5.2	49.42 ± 6.1	<0.001
Work support score (mean±SD)	65.08 ± 15.1	71.58 ± 13.8	61.26 ± 14.6	<0.001
Family support score (mean±SD)	32.62 ± 5.6	35.03 ± 4.9	31.20 ± 5.5	<0.001

**Table 2:** Descriptive characteristics of 2,986 pairs of propensity score matched participants.

Characteristic	Total	Burnout status		*p*
		Non-burnout	Burnout	
Age (N, %)				0.688
≤25	292(4.9)	136(4.6)	156(5.2)	
26–35	1,360(22.8)	668(22.4)	692(23.2)	
36–45	2049(34.3)	1,037(34.7)	1,012(33.9)	
46–55	2020(33.8)	1,018(34.1)	1,002(33.6)	
≥56	251(4.2)	127(4.3)	124(4.2)	
Gender (N, %)				0.379
Male	1973(33.0)	970(32.5)	1,003(33.6)	
Female	3,999(67.0)	2016(67.5)	1983(66.4)	
Office address (N, %)				0.052
Community	1915(32.1)	922(30.9)	993(33.3)	
Township	4,057(67.9)	2064(69.1)	1993(66.7)	
Education (N, %)				0.900
High school and below	1,409(23.6)	697(23.3)	712(23.8)	
Vocational school	2,584(43.3)	1,297(43.4)	1,287(43.1)	
Associate degree and above	1979(33.1)	992(33.2)	987(33.1)	
Professional title (N, %)				0.556
Intermediate and above	2,108(35.3)	1,074(36.0)	1,034(34.6)	
Entry	3,228(54.1)	1,597(53.5)	1,631(54.6)	
Senior	636(10.6)	315(10.5)	321(10.8)	
Working experience (N, %)				0.496
≤10 years	1,663(27.9)	811(27.2)	852(28.5)	
11 to 20 years	1,464(24.5)	739(24.7)	725(24.3)	
≥21 years	2,845(47.6)	1,436(48.1)	1,409(47.2)	
Annual income (N, %)				0.001
<30,000	987(16.5)	448(15.0)	539(18.1)	
30,000–50,000	2,301(38.5)	1,134(38.0)	1,167(39.1)	
>50,000	2,684(45.0)	1,404(47.0)	1,280(42.9)	
Daily working hours (N, %)				0.878
<8	2,998(50.2)	1,507(50.5)	1,491(49.9)	
8–10	2,227(37.3)	1,104(37.0)	1,123(37.6)	
>10	747(12.5)	375(12.6)	372(12.5)	
Overtime frequency (N, %)				0.621
No overtime required	1,223(20.5)	616(20.6)	607(20.3)	
Occasional overtime	3,483(58.3)	1724(57.7)	1759(58.9)	
Frequent overtime	1,266(21.2)	646(21.6)	620(20.8)	
Career Identity score (mean±SD)	51.90 ± 6.1	54.31 ± 5.2	49.49 ± 6.0	<0.001
Work support score (mean±SD)	66.80 ± 14.9	71.58 ± 13.8	62.02 ± 14.5	<0.001
Family support score (mean±SD)	33.19 ± 5.5	35.04 ± 4.9	31.34 ± 5.5	<0.001

### 3.2 Network comparisons

A comparison of network models between burnout and non-burnout populations after PSM based on NCT results showed that the global strength analysis reflected invariance (S = 0.148, *p* = 0.607), but the network structure showed significant differences (M = 0.261, *p* < 0.001), thus confirming H1.

### 3.3 Bridge nodes analysis

**Figure 1:**
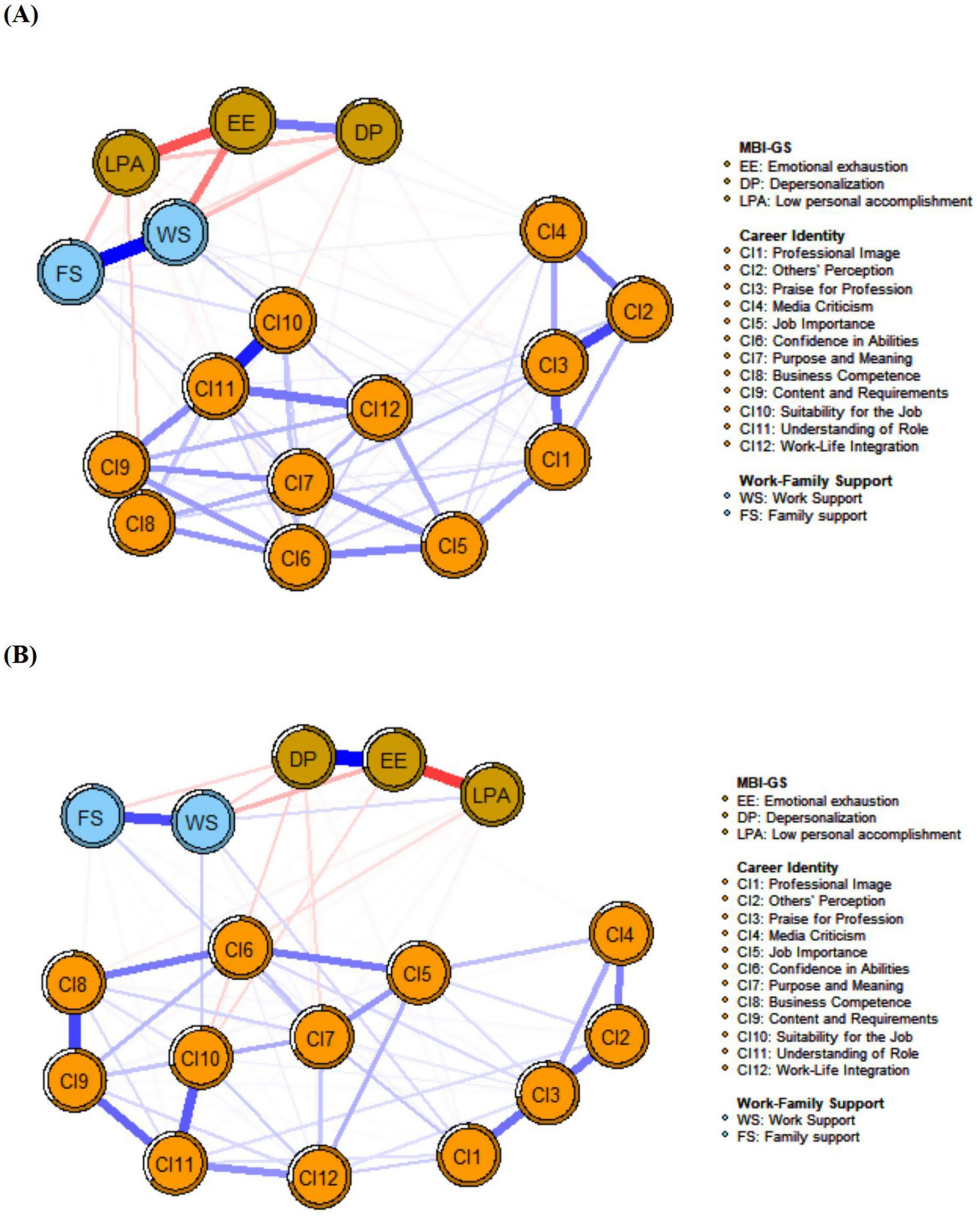
Network structures of the burnout-support-identity network for the burnout and non-burnout groups after PSM. **(A)** Network structure of non-burnout individuals after PSM. **(B)** Network structure of burnout individuals after PSM. Positive correlations are represented by blue edges, while negative correlations are shown with red edges. The thickness of an edge indicates the magnitude of the correlation, and the circles around nodes represent their predictability.

**Figure 2:**
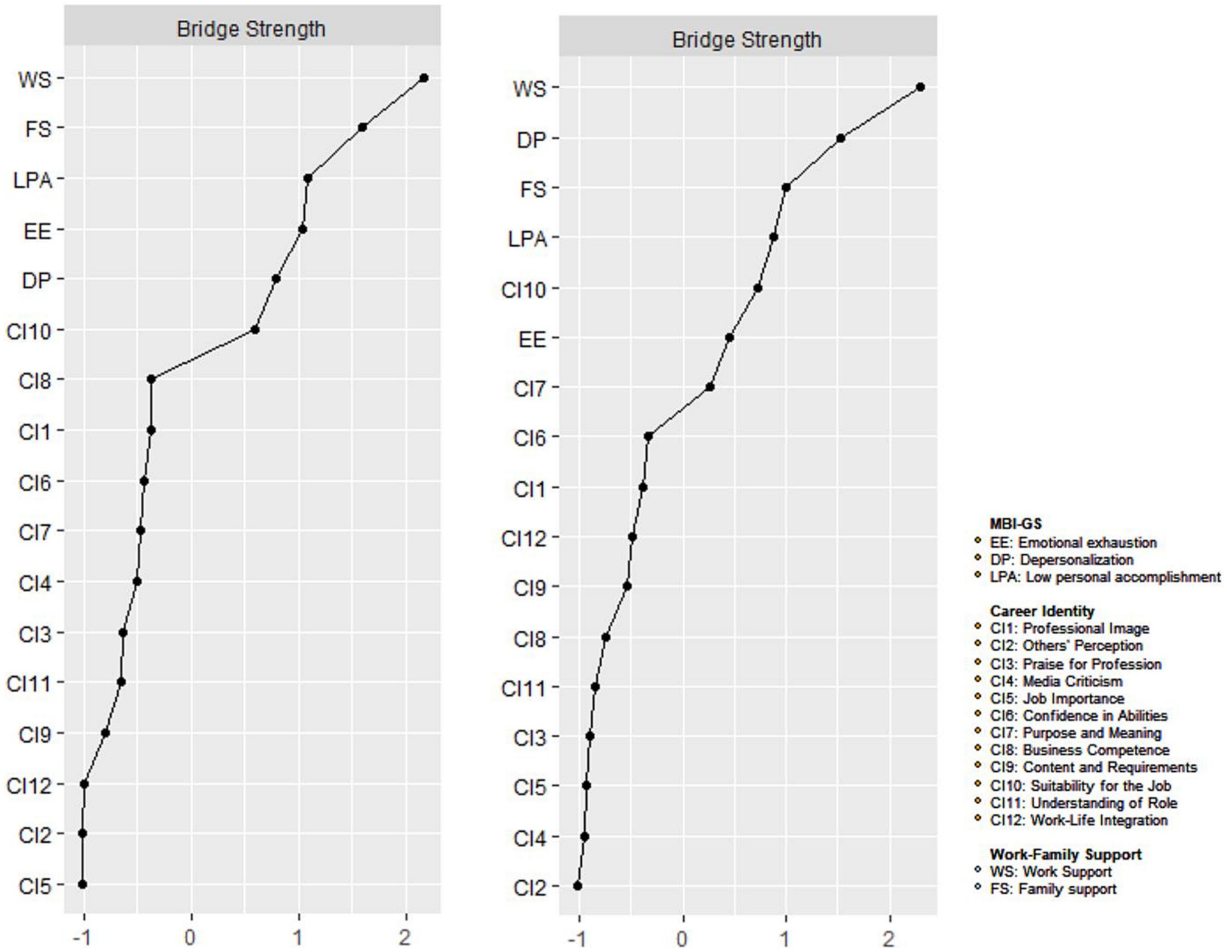
Centrality plot of the burnout-support-identity network for the burnout and non-burnout groups after PSM. Centrality plot depicting the strength and expected influence of each symptom in the burnout and non-burnout network (z-score) after PSM. The dash line represents the non-burnout individuals and solid line represent the burnout individuals.

Figure 1 illustrates the burnout-support-identity network model. Supplementary Figure S1 presents the bootstrap accuracy graph of the edge-weighted values of the network model. Within the cohort of non-burnout primary healthcare workers, WS (Work support) manifested as the most potent bridge node within the burnout-support-identity network. Subsequently, FS (Family support) and LPA (Low personal accomplishment) followed. In the context of burnout among primary healthcare workers, WS (Work support), followed by DP (Depersonalization), and FS (Family support), emerged as the most conspicuous bridge nodes (Figure 2), thus confirming H2.

### 3.4 Network structure and centrality nodes analysis

In the non-burnout population, the connection between CI8 (Business competence) and CI9 (Content and requirements) had the strongest positive advantage. As shown in Figure 1, the correlations between these nodes were not significant. However, the weighted values reveal their underlying connections, and the strongest positive dominant edges are presented in Supplementary Figure S1. Subsequently, we have the coupling of WS (Work support), FS (Family support), and the connection between CI10 (Suitability for the job), CI11 (Understanding of role). In the burnout population, the combination of EE (Emotional exhaustion), DP (Depersonalization) manifests the strongest positive advantage, followed by the association between CI8 (Business competence), CI9 (Content and requirements), and then WS (Work support), FS (Family support).

**Figure 3:**
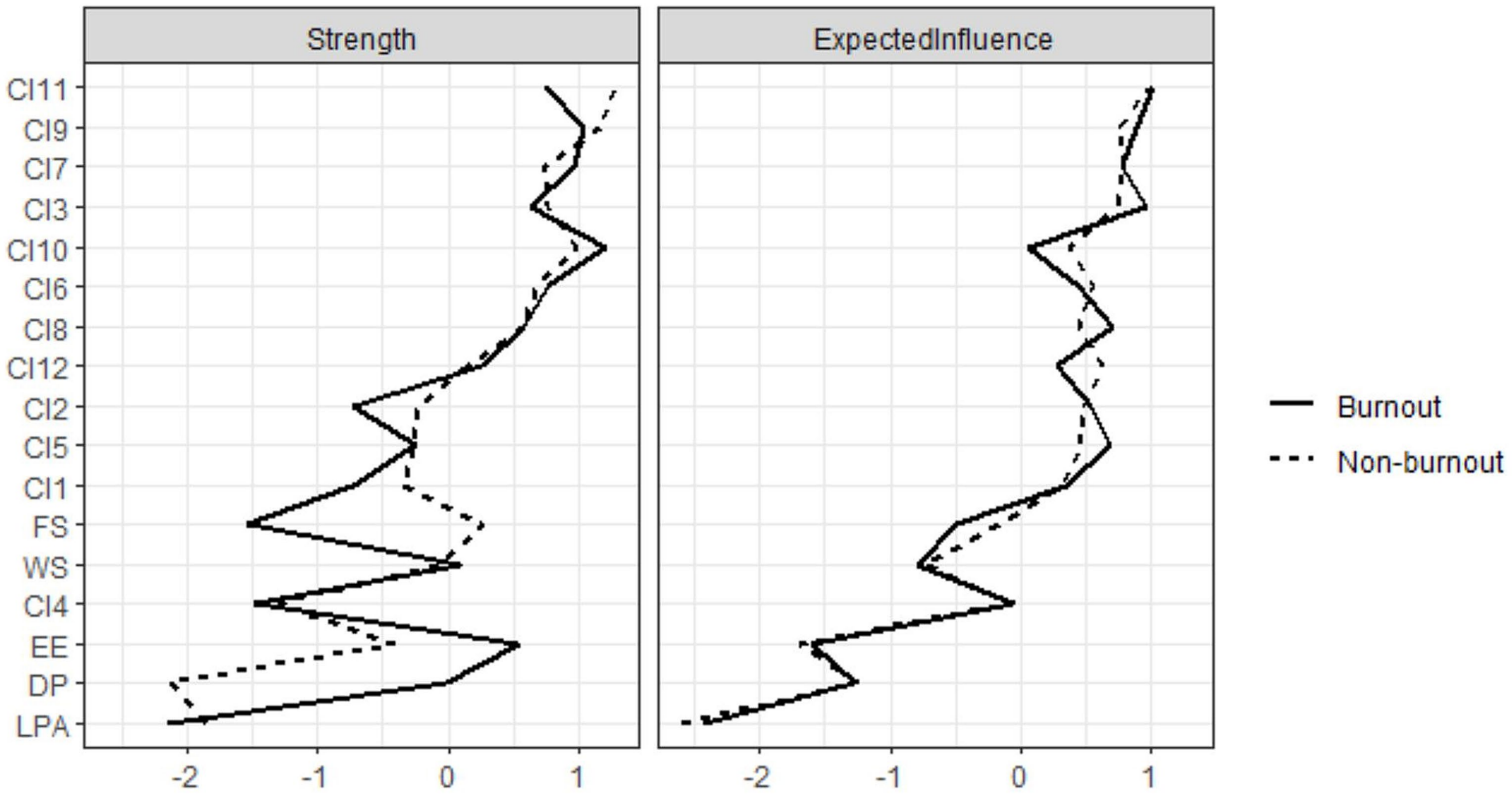
Bridge strength plot of the burnout-support-identity network for the burnout and non-burnout groups after PSM. Left panel: Index of bridge strength for each node of non-burnout group after PSM; Right panel: Index of bridge strength for each node of burnout group after PSM.

The network centrality nodes are shown in Figure 3. CI11 (Understanding of role) displayed the maximum EI (Expected influence) centrality within the network model of the non-burnout group. Subsequently, CI7 (Purpose and meaning) and CI9 (Content and requirements) followed in terms of EI centrality. In the network model of the burnout group, CI11 (Understanding of role) also emerged as the node with the highest EI centrality, followed by CI3 (Praise for professionals) and CI9 (Content and requirements). The detailed EI values are listed in Table 3.

**Table 3:** Descriptive statistics of measurement items.

Item content		Non-burnout after PSM				Burnout after PSM			
		M	SD	EI^*^	PRE	M	SD	EI^*^	PRE
Emotional exhaustion	EE	5.51	3.38	−0.30	0.88	10.42	4.71	−0.09	0.71
Depersonalization	DP	1.37	1.85	−0.12	0.92	5.94	3.90	0.11	0.75
Low personal achievement	LPA	7.54	6.69	−0.69	0.90	18.74	6.03	−0.52	0.85
Work support	WS	71.58	13.81	0.22	0.83	62.02	14.46	0.36	0.81
Family support	FS	35.04	4.88	0.44	0.84	31.34	5.46	0.45	0.85
Health workers’ professional image is my image.	OI1	4.54	0.64	0.74	0.80	4.12	0.73	0.80	0.77
I care a lot about how others perceive my profession.	OI2	4.32	0.84	0.74	0.83	3.99	0.85	0.79	0.79
Praising my profession is like praising me personally.	OI3	4.48	0.75	0.93	0.76	4.06	0.85	1.01	0.73
If the media criticizes my profession, I would feel embarrassed.	OI4	4.04	1.16	0.51	0.90	3.85	1.01	0.49	0.88
I consider my work important.	OI5	4.64	0.56	0.82	0.77	4.22	0.72	0.88	0.74
I have confidence in my work abilities.	OI6	4.67	0.50	0.90	0.69	4.24	0.63	0.94	0.67
I feel that the purpose of my work is clear and meaningful.	OI7	4.65	0.55	0.99	0.69	4.16	0.71	0.96	0.68
I possess the necessary professional skills.	OI8	4.57	0.56	0.86	0.64	4.18	0.64	0.84	0.67
I understand the content and requirements of my work.	OI9	4.63	0.53	1.00	0.61	4.25	0.60	1.01	0.63
The work I am currently engaged in suits me.	OI10	4.51	0.61	0.73	0.71	4.01	0.76	0.62	0.71
I understand my role.	OI11	4.58	0.55	1.10	0.63	4.15	0.63	1.04	0.65
My work is a part of my life.	OI12	4.67	0.53	0.89	0.72	4.25	0.68	0.79	0.72

M, mean score; SD, standard deviation; EI, expected influence; Pre, predictability; OI, occupational identity; EE, emotional exhaustion; DP, depersonalization; LPA, low personal achievement; WS, work support; FS, family support. *The values of the expected influence are raw data generated from the network.

Regarding network stability, as illustrated in Supplementary Figure S2, a high degree of stability was noted (CS-coefficient = 0.75 in EI and bEI), demonstrating that the network structure remained largely unchanged, even with the removal of 75% of the samples. Bootstrapped 95% confidence intervals suggest that the edge weights are reliable and stable. In addition, the bootstrap difference test revealed that most edge weight differences were statistically significant.

### 3.5 Flow network of job burnout

**Figure 4:**
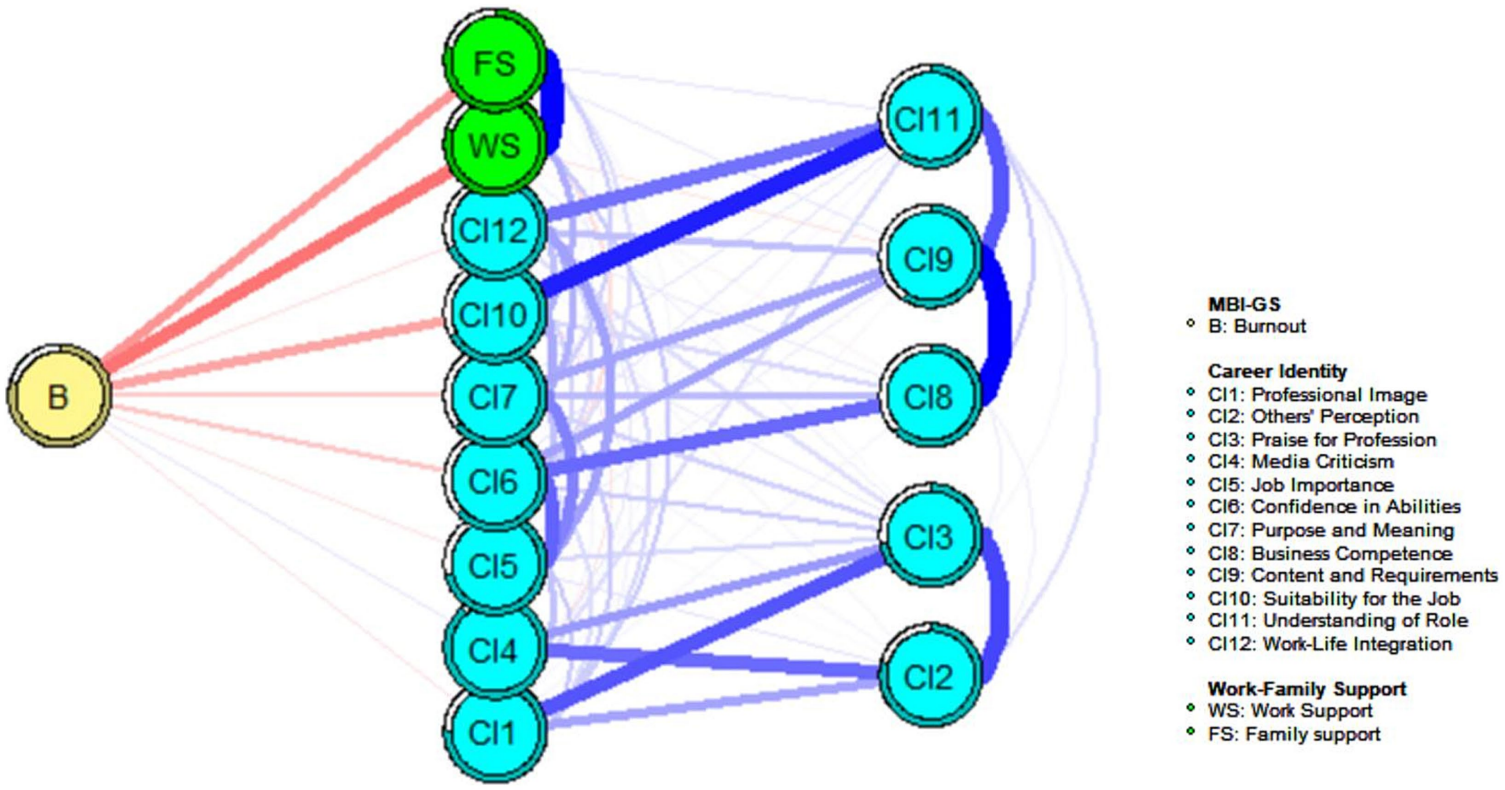
Flow network of burnout-support-identity in 2,986 pairs of propensity score matched participants. Blue edges represent positive partial correlations, and red edges represent negative partial correlations. Thicker edges represent stronger correlations.

Mobile networks highlight connectivity within the network model: The negative correlation connection between node B (Burnout) and WS (mean edge weight = − 0.21) is the strongest, followed by connections between node B (Burnout) and FS (mean edge weight = −0.16), and between node B (Burnout) and CI10 (Suitability for the job) (mean edge weight = −0.13). In addition, there was a positive correlation between CI4 (Media criticism) and Node B (Burnout) (mean edge weight = 0.05; Figure 4). The mechanism of occupational burnout was clarified through the network structure and flow network, further confirming hypothesis H3.

## 4 Discussion

In the 11th Revision of the International Classification of Diseases (ICD-11) the World Health Organization declared burnout an “occupational phenomenon” and acknowledged it as a serious health issue (36, 37). This is the first study to explore the interaction among burnout, career identity, and the structure of work-family support networks among Chinese primary healthcare workers from a network perspective. The burnout rate among primary healthcare workers was 62.9%, exceeding the burnout symptom incidence rate of over 50% among American physicians (38) but was lower than the burnout incidence rate of 79% among Canadian public health workers (39). This difference may reflect the characteristics of the different healthcare systems. Chinese primary healthcare workers have long faced the dual pressures of insufficient resources and high service demands, whereas North American studies have often focused on hospital system practitioners. It is worth noting that this study used the Chinese revised version of the Maslach Burnout Inventory, whose cultural adaptability may affect the comparison of results, which may partially explain the differences. Burnout has become an important issue affecting the mental health of healthcare workers globally (40). Therefore, it is necessary to develop interventions to address this issue. In this study, the scores of the burnout group in terms of career identity, work support, and family support were significantly lower than those of the non-burnout group, indicating that burnout is closely related to career identity and work-family support. This result is consistent with that of Yang et al. (27), who found that work-family support and career identity were negative predictors of burnout among primary healthcare workers.

CI11 (Understanding of role) and CI9 (Content and requirements) consistently emerged as central nodes in the burnout support–identity network of primary healthcare workers. This finding aligns with previous research indicating a significant correlation between role ambiguity and work stress among nurses (41, 42). For instance, Chinese nurses who often work over 50 h a week and have frequent night shifts are prone to “defensive pessimism, “a coping mechanism through which they alleviate anxiety by anticipating the worst outcomes, such as medical errors. This outlook further undermines their professional identity. In contrast, Vietnam has successfully reduced the turnover intention of primary healthcare workers by 18% through “job readiness training,” which clarifies the boundaries of responsibilities and demonstrates the protective effect of role clarity on professional identity. Weehoe et al. (43) found that, during a public health crisis, insufficient understanding of work content and requirements among medical staff increased physical fatigue and psychological stress. Therefore, China should focus on strengthening the institutionalization of job responsibilities by clarifying service lists and assisting healthcare workers in reconstructing their sense of professional meaning through narrative therapy.

The central node CI3 (Praise for professionals) had a stronger impact on the network of primary healthcare workers experiencing burnout than on those without burnout, indicating its importance in the burnout network. Morera et al. (44) found that praise and admiration for healthcare professionals’ dedication can help them overlook burnout and distress. Schaeffer (45) discovered that the burnout level of healthcare professionals was related to praise and suggested increasing interventions to reduce the level of burnout. “Praise for professionals” was negatively correlated with the severity of burnout, suggesting that providing sufficient praise and emotional value can serve as an effective intervention (46). However, in the burnout network of primary healthcare workers, the influence of DP (depersonalization) is stronger than in the non-burnout network. “Depersonalization” refers to the process by which an individual loses self-awareness and career identity sense in a group situation (47). Onder et al. (48) showed that emotional exhaustion and depersonalization were coupled and found that nurses with a higher degree of emotional exhaustion reported a higher level of depersonalization. Altinay (49) found that career identity has a strong negative correlation with depersonalization and exhaustion, suggesting customized support measures to overcome burnout. Therefore, primary health care workers should consider developing and strengthening their career identities and enhancing interventions targeting depersonalization to improve their mental health and reduce burnout.

The bridge nodes consistent in the burnout and non-burnout networks of primary healthcare workers are WS (Work support) and FS (Family support). This indicates that WS and FS are significantly correlated with career identity and burnout. This support may be associated with enhanced career identity and reduced burnout. However, further research is needed to establish causality. Jiang et al. (50) found that career identity and emotional exhaustion of primary care physicians were significantly related to work-family support. This finding resonates with the conclusion of Jiang et al. (27): for every unit increase in work–family conflict among Chinese primary care doctors, the risk of emotional exhaustion rises by 37%. Notably, North American studies often emphasize how “flexible work arrangements” improve family support, whereas the challenges faced by Chinese primary healthcare workers are more related to administrative tasks encroaching on core service time (51, 52). In response, the “trinity” intervention model could serve as a useful reference 1. In-hospital psychological support stations and emergency rooms should be established. 2. Implementing “family open days” to promote family understanding. 3. Utilizing digital tools to streamline administrative processes increased the time that healthcare workers spent on patient services from 58 to 72%. In addition, providing more work-and family related support to primary care workers can enhance their professional identities and reduce burnout.

The job burnout flu network shows that work-family support has the greatest negative correlation with job burnout, followed by CI10 (Suitability for the job). Di Trani et al. (53) found that the personal resilience level of medical staff prevented burnout and proposed the importance of the targeted enhancement of personal skills. In their study of healthcare professionals, Bridgeman et al. (54) found that inconsistencies in a person’s personality and work environment can lead to burnout syndrome. In addition, a positive correlation was observed between job burnout and CI4 (Media criticism). Wilson et al. (55) found that risk factors for depersonalization included facing more criticism and identified criticism as one of the predictors of high levels of burnout among doctors and nurses. According to Nunes (56), criticism from the outside world can affect nurses’ health and lead to burnout, and self-control over criticism is a predictor of nurse burnout.

Network analysis has been used to examine various mental disorders including depression, anxiety, psychosis, autism spectrum disorders, substance use disorders, posttraumatic stress disorder, insomnia, and other mental health symptoms and behaviors (57–60). The focus of this study on job burnout, career identity, and work-family support highlights a range of psychological issues, including stress and professional dissatisfaction, although our study did not specifically address the relationship between burnout and other common psychological health issues such as depression. Previous studies have suggested that various intricate mechanisms are involved in the effect of work-related stress on mental health. Negative emotions such as burnout and anxiety may be triggered by factors commonly associated with online social issues, including social comparison and self-evaluation, social pressures and loneliness, information overload, and exposure to negative content (61–63). According to our network research, specific factors such as a lack of understanding of roles and ambiguity in job requirements exacerbate feelings of uncertainty and job burnout among primary healthcare workers in high-pressure work environments, particularly affecting the mental health of those lacking work and family support. Analyzing the relationship between these factors and job burnout not only aids in the development of more effective intervention strategies to promote healthier and more sustainable work environments for primary healthcare workers but also sheds light on the potential role that work-family support and career identity may play in the emergence and progression of mental health issues, particularly symptoms of stress and professional dissatisfaction triggered by burnout-related challenges.

### 4.1 Strengths and limitations

This study revealed the complex interrelationships between job burnout, career identity, and work-family support among primary healthcare workers through network analysis. Furthermore, it identified network differences between burnout and non-burnout groups and highlighted the central and bridge nodes, providing a theoretical basis for formulating targeted intervention measures. However, this study had several limitations. First, because the data used in this study were cross-sectional, it was not possible to determine the direction of the network edges; thus, causal relationships between symptoms could not be established. Second, owing to the nature of burnout, the assessment of identity and work-family support relied on the participants’ self-reports, which indicates the possibility of response bias in the data. Third, the study did not include general mental health assessment tools to control for or examine potential confounding factors related to mental health disorders such as depression and anxiety. The failure to consider these factors may have affected the interpretation of our results. Finally, as all participants were Chinese, the generalizability of these findings to other countries may be limited, and further studies are required to validate these results in different populations and countries. Further research is required to verify the applicability of these results to different countries.

## 5 Conclusion

In conclusion, this study found that the central nodes in the burnout-support-identity network of primary healthcare workers were CI11 (Understanding of role) and CI9 (Content and requirements), and the bridge nodes were WS (Work support) and FS (Family support). Burnout was closely related to WS (Work support), FS (Family support), CI10 (Suitability for the Job), and CI4 (Media criticism). To alleviate job burnout, targeted prevention and intervention strategies must be developed with a focus on core and bridge nodes to support the mental health of primary healthcare workers, improve the quality of primary healthcare services, and enhance the overall effectiveness of the healthcare system.

## Data Availability

The original contributions presented in the study are included in the article/supplementary material, further inquiries can be directed to the corresponding authors.
