# Supplementary material for: Network analysis of work-family support and career identity and their associations with job burnout among primary healthcare workers: a cross-sectional study
Source: Front Public Health. 2025 Jun 26;13:1581624. doi: 10.3389/fpubh.2025.1581624 (PMC12241083; doi:10.3389/fpubh.2025.1581624)
Supplement: Supplementary file 1 [file Supplementary_file_1.docx]

**A. B.**

**
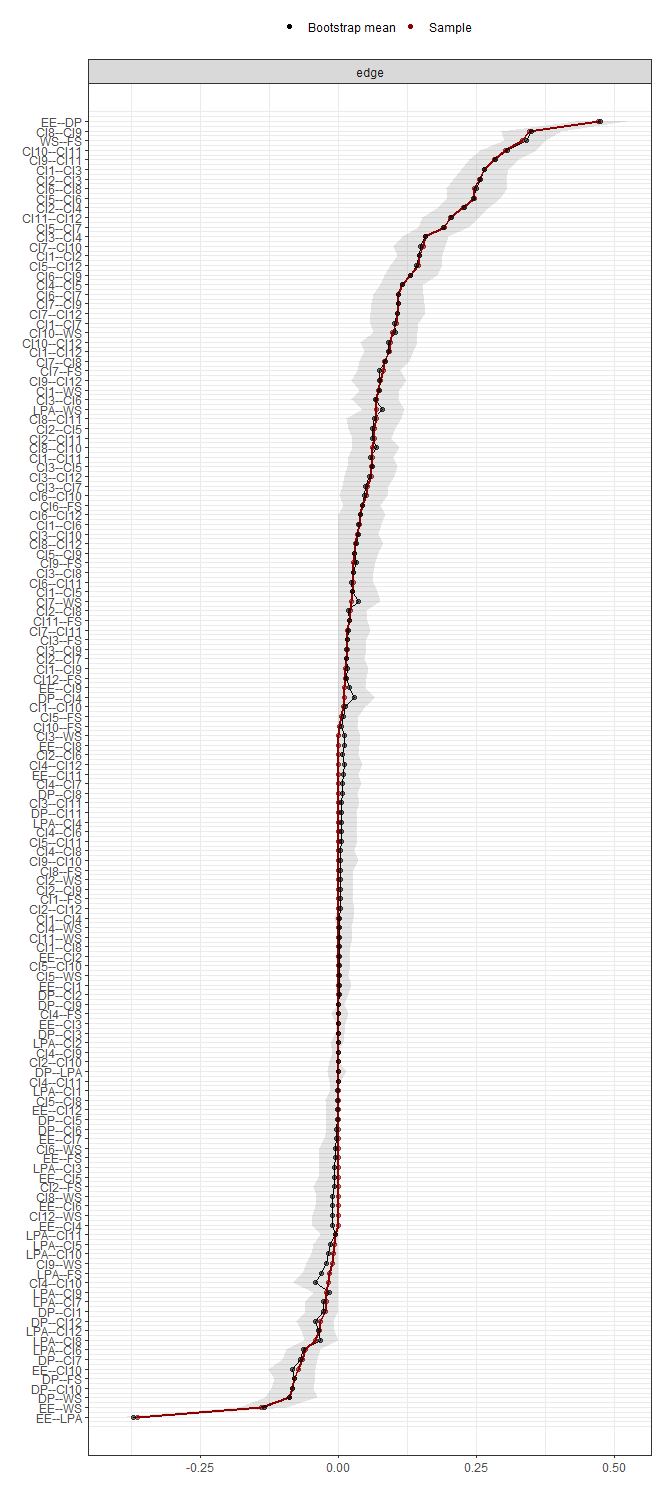
**
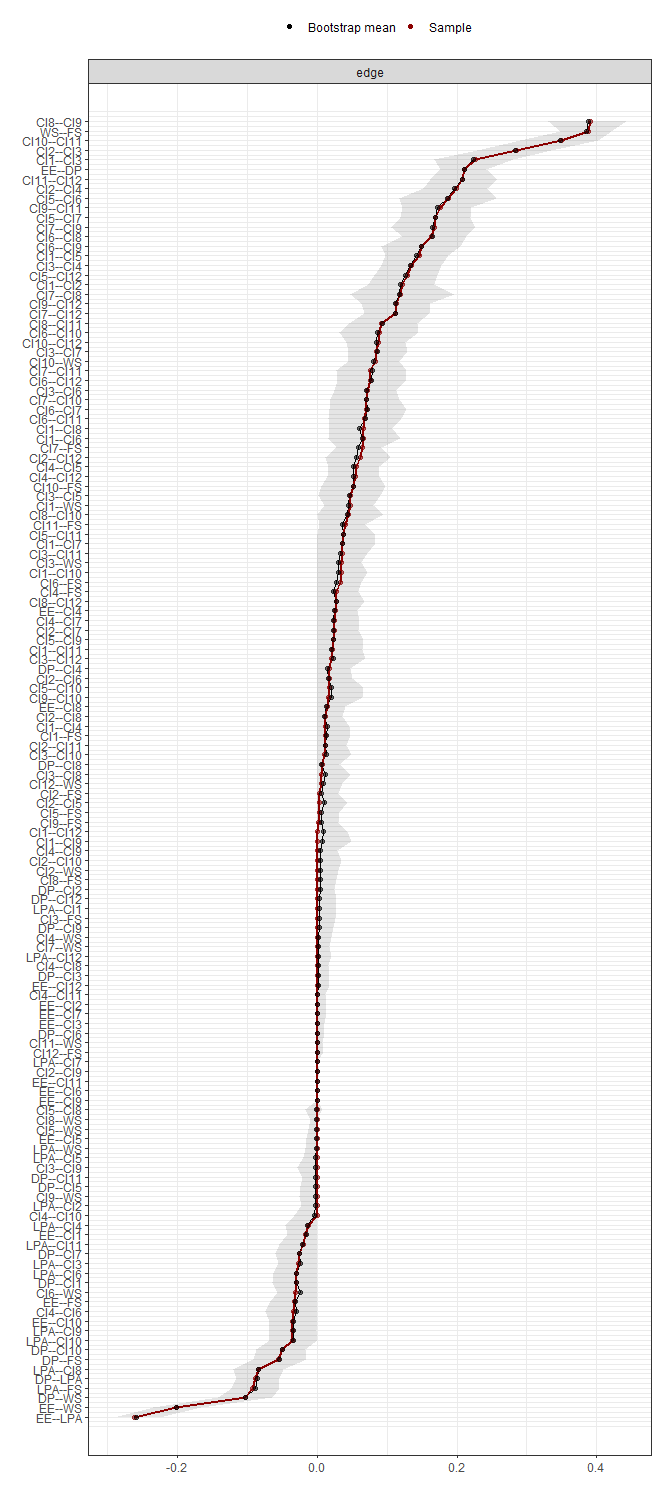


**Fig S1. Accuracy of edge weights.** **A.** bootstrapped accuracy of edge weight in the burnout-support-identity network of non-burnout populations after PSM. **B.** bootstrapped accuracy of edge weight in the burnout-support-identity network of burnout populations after PSM.The red line shows edge weights and the gray bar shows the confidence interval.


**
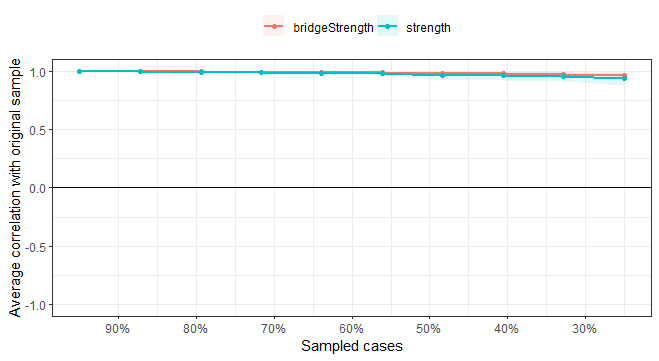
B.
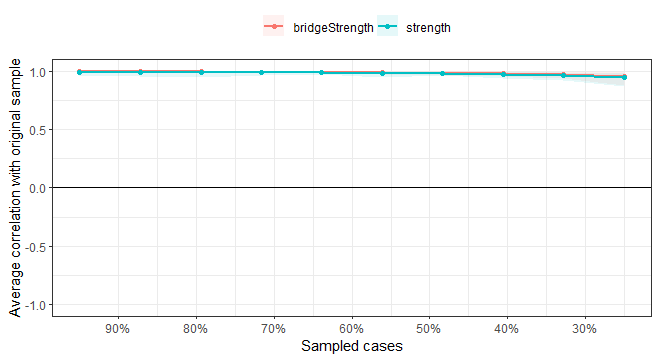
**

**Fig S2. Stability of node strength and expected influences. A.** bootstrapped stability of node strength and expected influences in the burnout-support-identity network of non-burnout populations after PSM. **B.** bootstrapped stability of node strength and expected influences in the burnout-support-identity network of burnout populations after PSM.The red bar shows the average correlation between the full sample and subsample for the metrics of node strength and expected influences. The red area displays the range from the 2.5th quantile to the 97.5th quantile.
